# Pharmacological treatment of schizophrenia: Japanese Expert Consensus 2025

**DOI:** 10.1038/s41537-026-00770-x

**Published:** 2026-06-02

**Authors:** Yoshiteru Takekita, Hideaki Tani, Yasushi Kawamata, Asuka Katsuki, Hiroyuki Muraoka, Shinichiro Ochi, Hiromi Tagata, Kyosuke Sawada, Akira Nishi, Saki Hattori, Toshiaki Kikuchi, Hajime Baba, Masaki Kato, Koichiro Watanabe, Ken Inada, Norio Yasui-Furukori, Hitoshi Sakurai, Jun-ichi Iga

**Affiliations:** 1 Department of Neuropsychiatry, Faculty of Medicine, Kansai Medical University, Osaka, Japan; 2 Department of Neuropsychiatry, Keio University School of Medicine, Tokyo, Japan; 3 Department of Psychiatry, Dokkyo Medical University School of Medicine, Tochigi, Japan; 4 Nijoufukushikai Social Welfare Corporation, Fukuoka, Japan; 5 Department of Psychiatry, Kitasato University School of Medicine, Kanagawa, Japan; 6 Department of Neuropsychiatry, Molecules and Function, Ehime University Graduate School of Medicine, Ehime, Japan; 7 Department of Neuropsychiatry, Toho University Faculty of Medicine, Tokyo, Japan; 8 Department of Psychiatry, Yokohama City University School of Medicine, Kanagawa, Japan; 9 Department of Psychiatry & Behavioral Science, Juntendo University Graduate School of Medicine, Tokyo, Japan; 10 Department of Neuropsychiatry, Kyorin University School of Medicine, Tokyo, Japan

**Keywords:** Schizophrenia, Psychosis

## Abstract

Conventional schizophrenia treatment guidelines do not adequately address all clinically important issues in routine practice. This study aimed to update the 2021 expert consensus of the Japanese Society of Clinical Neuropsychopharmacology (JSCNP) to reflect the current clinical landscape. A total of 154 board-certified psychiatrists from the JSCNP and the Japanese Society of Neuropsychopharmacology (JSNP) evaluated treatment options across 21 clinically relevant situations using a 9-point Likert scale (1 = “strongly disagree”; 9 = “strongly agree”); the response rate was 44%. First-line antipsychotics varied by predominant symptoms: risperidone, brexpiprazole, olanzapine, paliperidone, and blonanserin for positive symptoms; aripiprazole and brexpiprazole for negative symptoms and cognitive impairment; aripiprazole, brexpiprazole, lurasidone, olanzapine, and quetiapine for depression and anxiety; brexpiprazole, aripiprazole, and olanzapine for disorganized thinking; olanzapine and risperidone for excitement and aggression; and aripiprazole, brexpiprazole, and lurasidone for social integration. Brexpiprazole, quetiapine, and aripiprazole were first-line options for patients at high risk of extrapyramidal side effects or diabetes mellitus. Dose reduction or switching was the treatment of choice for tardive dyskinesia. Repeated recurrence, patient request, and poor medication adherence were indications for introducing long-acting injectable antipsychotics. Switching to clozapine was the treatment of choice for treatment-resistant schizophrenia. Adverse effects were the highest-rated factor for both dose reduction and simplification to antipsychotic monotherapy. Second-generation antipsychotics were rated as first- or second-line options in most situations, whereas first-generation antipsychotics were generally rated as third-line. These recommendations provide practical guidance for treatment selection and shared decision-making in clinically challenging situations not adequately addressed by existing evidence alone.

## Introduction

Several clinical practice guidelines have been published to improve the quality of care and treatment outcomes in schizophrenia^1–4^. These guidelines describe comprehensive treatment approaches based mainly on randomized controlled trials (RCTs) and subsequent meta-analyses, including evidence on the efficacy and adverse effects of antipsychotics. However, the strict inclusion and exclusion criteria used in RCTs limit generalizability, and the available evidence may not address all clinical challenges encountered in daily practice. Expert consensus based on frontline clinical experience is therefore useful for filling these gaps and supporting shared decision-making (SDM) in real-world practice.

The Japanese Society of Clinical Neuropsychopharmacology (JSCNP) is an academic society dedicated to clinical psychopharmacology for psychiatric disorders and, together with the Japanese Society of Neuropsychopharmacology (JSNP), jointly certifies specialist psychiatrists based on academic achievement and written examinations. The JSCNP published its first expert consensus on the pharmacological treatment of schizophrenia in 2021^5^, but an updated consensus is now needed because the clinical landscape in Japan has changed substantially since the 2019 survey.

Since the previous survey, treatment options have become more complex with the introduction of lurasidone and the wider use of second-generation antipsychotics (SGAs). Formulations have also diversified to include long-acting injectable (LAI) antipsychotics and transdermal patches. In addition, vesicular monoamine transporter 2 (VMAT2) inhibitors for tardive dyskinesia (TD) have emerged, and monitoring of clozapine (CLO) plasma levels is now reimbursed under the national health insurance system. Safety and tolerability are also increasingly prioritized, with growing interest in dose reduction and simplification to antipsychotic monotherapy.

We therefore conducted a new survey of certified psychiatrists from the JSCNP and JSNP to update the previous consensus in line with current practice. The study aimed to establish expert consensus in Japan on 21 clinical scenarios in the treatment of schizophrenia with limited evidence, including symptom-based pharmacotherapy, criteria for LAI initiation, management of adverse effects, agitation or medication refusal, treatment resistance, and dose reduction or simplification to antipsychotic monotherapy.

## Methods

### Study design

This survey was conducted between September 16 and October 17, 2025. Following the approach used in the 2019 expert consensus^5^, an 18-member working group led by the Medical Education Committee of the JSCNP identified 21 clinical situations and corresponding questions after reviewing current clinical practice guidelines for schizophrenia. For each situation, the working group developed a specific question and a corresponding set of treatment options. A total of 350 psychiatrists holding specialist certification in neuropsychopharmacology, jointly granted by the JSCNP and the Japanese Society of Neuropsychopharmacology (JSNP), were invited by email to participate in the survey.

Participants who agreed to take part rated the proposed options on a 9-point Likert scale (1 = “strongly disagree”; 9 = “strongly agree”). The clinical situations and treatment options are listed in Supplementary Table 1. Respondents were instructed to assign a score of 9 to at least one option if they endorsed any listed treatment choice and a score of 1 to all options if they endorsed none. The survey required approximately 15–30 min to complete. Participation was voluntary, and no incentives were provided. This study was approved by the Institutional Review Board of Ehime University Hospital (approval number: 2511004). As the survey was anonymous and voluntary, completion of the questionnaire was considered to constitute implied consent. The board waived the requirement for written informed consent. Respondents also provided information on years of clinical experience, sex, and work location.

### Analysis

All statistical analyses were performed using IBM SPSS Statistics version 25 (IBM Corp., Armonk, NY, USA). For each treatment option, the mean, standard deviation (SD), 95% confidence interval (CI), and numbers of responses in the 1–3 (disagree), 4–6 (neutral), and 7–9 (agree) ranges were calculated. Pearson’s chi-squared test was used to compare response frequencies across the three rating categories (disagree, neutral, and agree) for each treatment option. When responses did not differ significantly across the three rating categories (p ≥ 0.05), the option was classified as showing no consensus, consistent with previous reports^5,6^. Treatment options with a lower bound of the 95% CI of ≥6.5 were classified as first-line, those with a lower bound of the 95% CI of ≥3.5 as second-line, and the remaining options as third-line. Options rated 9 by more than 50% of respondents were defined as treatments of choice.

In general, first-line treatment refers to the intervention considered most appropriate as the initial approach, or the factor regarded as most important, in a given situation^7^. A treatment of choice represents a particularly strong first-line recommendation. Second-line treatment refers to a reasonable option for patients who do not respond to or cannot tolerate first-line treatment, or the next most important option after first-line options. No-consensus treatment refers to a controversial strategy or factor. Third-line treatment refers to an option generally regarded as inappropriate and used only when preferred alternatives have failed. For decision factors, third-line refers to one that is not usually considered important.

## Results

### Participant characteristics

Of the 350 invited certified psychiatrists, 154 completed the questionnaire (response rate, 44.0%). Mean clinical experience was 25.2 ± 10.2 years. Affiliations were university hospitals (57, 37.0%), psychiatric hospitals (47, 30.5%), community clinics (30, 19.5%), general hospitals (10, 6.5%), and other institutions (10, 6.5%).

### Pharmacological treatment selection based on predominant psychiatric symptoms (Q1–Q7)

First-line treatment selection varied across symptom domains (Table 1; Supplementary Figs. 1–7). For positive symptoms (Q1), risperidone (RIS), brexpiprazole (BRE), olanzapine (OLA), paliperidone (PAL), and blonanserin (BLO, including the transdermal patch) were first-line. For negative symptoms (Q2), aripiprazole (ARI) and BRE were first-line. For depression and anxiety (Q3), ARI, BRE, lurasidone (LUR), OLA, and quetiapine (QUE) were first-line. For excitement and aggression (Q4), OLA and RIS were first-line. For disorganized thinking (Q5), BRE, ARI, and OLA were first-line. For cognitive impairment (Q6), BRE and ARI were first-line. For social integration in patients without noticeable symptoms (Q7), ARI, BRE, and LUR were first-line.

**Table 1:** Consensus on the choice of antipsychotics depending on predominant symptoms.

	Positive symptoms	Negative symptoms	Depression/anxiety	Excitement/aggression	Disorganized thinking	Cognitive impairment	Social integration
Aripiprazole	1st	1st	1st	2nd	1st	1st	Treatment of choice
Brexpiprazole	1st	1st	1st	2nd	1st	1st	Treatment of choice
Olanzapine	1st	2nd	1st	Treatment of choice	1st	2nd	2nd
Risperidone	1st	2nd	2nd	1st	2nd	2nd	2nd
Lurasidone	2nd	2nd	1st	2nd	2nd	2nd	1st
Blonanserin							
(including transdermal patch)	1st	2nd	2nd	2nd	2nd	2nd	2nd
Paliperidone	1st	2nd	2nd	2nd	2nd	2nd	2nd
Quetiapine	2nd	2nd	1st	2nd	2nd	2nd	2nd
Asenapine	2nd	2nd	2nd	2nd	2nd	2nd	2nd
Perospirone	2nd	2nd	2nd	2nd	2nd	2nd	2nd
Clozapine	2nd	2nd	no consensus	2nd	2nd	2nd	no consensus
Zotepine	3rd	3rd	3rd	2nd	3rd	3rd	3rd
Perphenazine	3rd	3rd	3rd	3rd	3rd	3rd	3rd
Fluphenazine	3rd	3rd	3rd	3rd	3rd	3rd	3rd
Sulpiride	3rd	3rd	3rd	3rd	3rd	3rd	3rd
Chlorpromazine	3rd	3rd	3rd	no consensus	3rd	3rd	3rd
Levomepromazine	3rd	3rd	3rd	no consensus	3rd	3rd	3rd
Haloperidol	no consensus	3rd	3rd	no consensus	3rd	3rd	3rd

Treatment-line categories were defined based on the lower bound of the 95% confidence interval (CI) of the mean score: options with a lower CI bound of ≥6.5 were classified as first-line; those with a lower CI bound of ≥3.5 as second-line; and the remainder as third-line. Options rated 9 by more than 50% of respondents were defined as treatments of choice. When responses were evenly distributed across the three rating categories (disagree, 1–3; neutral, 4–6; agree, 7–9), the option was classified as showing no consensus (*p* ≥ 0.05, Pearson’s chi-squared test).

*SGA* second-generation antipsychotic, *FGA* first-generation antipsychotic.

OLA for Q4 and ARI and BRE for Q7 were the treatments of choice. Across Q1–Q7, other SGAs were generally second-line, except CLO, which was classified as no consensus for Q3 and Q7. Most first-generation antipsychotics (FGAs) were third-line. Exceptions classified as no consensus were haloperidol (HAL) for Q1 and chlorpromazine, levomepromazine (LEV), and HAL for Q4.

### Treatment selection in the presence of adverse events (Q8–Q10)

For patients at high risk of extrapyramidal side effects (EPS) (Q8), QUE, BRE, and ARI were first-line. All other SGAs were second-line, and all FGAs were third-line (Supplementary Fig. 8).

For patients with diabetes mellitus (Q9), BRE, ARI, LUR, and BLO, including the transdermal patch, were first-line. BRE and ARI were the treatments of choice. FGAs, QUE, and OLA were third-line, whereas the remaining SGAs were second-line (Supplementary Fig. 9).

For tardive dyskinesia (TD) management (Q10), dose reduction and switching were first-line treatments of choice. Valbenazine and electroconvulsive therapy (ECT) were second-line. Clonazepam (off-label) was classified as no consensus, and all other treatments were third-line (Supplementary Fig. 10).

### Selection of long-acting injectable antipsychotics (Q11–Q12)

Repeated recurrence, patient request, and poor medication adherence were situations in which LAI initiation (Q11) was rated as the treatment of choice (Supplementary Fig. 11).

For LAI selection (Q12), tolerability, efficacy, prior LAI treatment history, current severity of positive symptoms, and antipsychotic class (FGA vs SGA) were first-line. Tolerability and efficacy were the factors of choice, and all remaining factors were second-line (Supplementary Fig. 12).

### Treatment selection for agitation and medication refusal in specific clinical situations (Q13–Q15)

For concomitant antipsychotic use when agitation persisted despite monotherapy (Q13), OLA and RIS were first-line. Zotepine, LEV, BRE, ARI, chlorpromazine, and HAL were classified as no consensus (Supplementary Fig. 13).

For pro re nata (PRN) use in temporarily intensified agitation (Q14), RIS oral solution, OLA intramuscular injection, and OLA orally disintegrating tablet were first-line. HAL intramuscular and intravenous injections, BLO transdermal patch, LEV intramuscular injection, lorazepam, LEV, ARI oral solution, and chlorpromazine were classified as no consensus (Supplementary Fig. 14).

For patients with persistent medication refusal (Q15), the BLO transdermal patch was the only first-line option. RIS LAI and HAL intramuscular and intravenous injections were classified as no consensus (Supplementary Fig. 15).

### Treatment selection related to treatment-resistant schizophrenia (TRS) (Q16–Q19)

For TRS (Q16), switching to CLO was the treatment of choice, and concomitant ECT was first-line. ECT monotherapy was classified as no consensus, and all other treatments were third-line (Supplementary Fig. 16).

For first-episode TRS requiring physical restraint (Q17), no first-line consensus was reached regarding the number of antipsychotics to try before CLO initiation. All options were second-line (Supplementary Fig. 17).

Similarly, for outpatient first-episode TRS (Q18), no first-line consensus was reached, and all options were second-line (Supplementary Fig. 18).

For clozapine-resistant schizophrenia (CRS) (Q19), concomitant ECT, waiting more than 8 weeks for a response, and increasing the CLO dose to more than 200 mg were first-line. Concomitant mood stabilizer use and discontinuation of CLO followed by ECT monotherapy were classified as no consensus, and all other treatments were third-line (Supplementary Fig. 19).

### Factors to consider when pursuing dose reduction or simplification to antipsychotic monotherapy (Q20–Q21)

For dose reduction (Q20), first-line factors were adverse effects, patient understanding of relapse prevention, ability to recognize early signs of deterioration, good current social adaptation, insight into illness, clinical stability for more than 6 months, mild residual positive symptoms, mild residual excitement and aggression, and desire for future pregnancy in female patients. Adverse effects were the highest-priority factor, a high number of past episodes were classified as no consensus, and all other factors were second-line (Supplementary Fig. 20).

For simplification to monotherapy (Q21), first-line factors were adverse effects, mild residual positive symptoms, patient understanding of relapse prevention, patient preference, mild residual excitement and aggression, good current social adaptation, ability to recognize early signs of deterioration, clinical stability for more than 6 months, and insight into illness. Additional first-line factors were recommendations from clinical practice guidelines, desire for future pregnancy in female patients, no history of self-harm or harm to others, LAI use to facilitate monotherapy, family preference for monotherapy, good past treatment response, and mild residual depressive and anxiety symptoms. Adverse effects were the highest-priority factor, a high number of past episodes were classified as no consensus, and all other factors were second-line (Supplementary Fig. 21).

## Discussion

This survey evaluated pharmacological treatment recommendations for clinical situations not adequately addressed by existing guidelines.

For symptom-based antipsychotic selection (Q1–Q7), all SGAs were categorized as first- or second-line, whereas FGAs were third-line or classified as no consensus in all scenarios. Compared with the previous survey^5^, the main changes were the elevation of BRE, PAL, BLO, and LUR to first-line treatment status.

BRE and ARI were highly rated across multiple scenarios, except for excitement and aggression. Network meta-analyses of both global, multi-ethnic cohorts and Japanese-specific populations suggest that, while no major differences in efficacy exist among SGAs (excluding clozapine), these D2 partial agonists consistently demonstrate a favorable tolerability profile—specifically lower risks of EPS and metabolic side effects^8–10^. This pattern suggests that Japanese specialists place greater emphasis on tolerability than on efficacy, consistent with recent international guidelines that support SDM based on adverse-effect profiles^11^. The present survey did not identify clear criteria for differentiating between ARI and BRE.

The elevation of PAL and BLO to first-line status for positive symptoms may reflect both favorable meta-analytic findings and the diversification of available formulations, particularly PAL LAI and the BLO transdermal patch introduced in 2019^8,12^. In the social integration scenario, LUR was newly categorized as first-line, possibly reflecting benefits for social functioning and a favorable metabolic profile^8,13^.

The newly added scenarios of disorganized thinking and cognitive impairment yielded results similar to those for negative symptoms. These symptom dimensions may share a common feature of broad cognitive dysfunction^14,15^. This pattern may indicate a shared therapeutic approach favoring activating D2 partial agonists with relatively benign adverse-effect profiles.

FGA endorsement declined further than in the previous survey, possibly because accumulating evidence on EPS risk and dose-dependent gray matter volume reduction has reduced their use in real-world practice^8,16^.

In adverse-event scenarios (Q8–Q10), most SGAs were categorized as first- or second-line for high EPS risk and diabetes mellitus. For TD management, dose reduction or switching was first-line, whereas valbenazine and ECT were second-line, and most other options were third-line.

Recommendations for patients at high EPS risk (Q8) were largely consistent with the previous survey, except for elevating BRE to first-line. The findings more closely resembled international guidelines and the Maudsley Prescribing Guidelines than network meta-analytic rankings^8,11,17^, favoring D2 partial agonists or agents with lower D2 receptor affinity.

A similar pattern was observed for diabetes mellitus (Q9). QUE and OLA, which are contraindicated in diabetic patients in Japan, were third-line^18^. Rankings of other agents also diverged somewhat from network meta-analytic estimates of glucose-elevation risk^18^ and more closely reflected the clinical priorities emphasized in the Maudsley Prescribing Guidelines^17^. These findings suggest that Japanese specialists do not rely solely on quantitative data but also weigh the overall risks and benefits for individual patients.

TD management (Q10) warrants three brief comments. First, dose reduction and switching were first-line, despite network meta-analyses and American Academy of Neurology guidelines raising concerns about symptom worsening and insufficient evidence^19,20^. This likely reflects a pragmatic approach aligned with European guidelines that prioritize avoidance of harm and polypharmacy^21^. Second, valbenazine, despite having the strongest evidence base among available TD treatments^4,19,20,22^, was only second-line, likely because clinicians also considered cost-effectiveness and tolerability. Third, ECT was second-line despite an evidence base consisting mainly of case reports^23^, suggesting that specialists still view it as a rescue option for severe refractory cases. Overall, this consensus prioritizes the removal of causative factors and reserves VMAT2 inhibitors and ECT for refractory cases.

For LAI introduction and selection (Q11–Q12), LAI initiation was the treatment of choice for repeated recurrence, patient request, and poor adherence. For LAI selection, tolerability and efficacy were the highest-priority factors, and prior LAI treatment history, current severity of positive symptoms, and FGA/SGA distinction were also first-line factors.

LAI utilization in Japan is estimated at 5–10% among patients with schizophrenia^24,25^. The endorsement of repeated recurrence, patient request, and poor adherence is consistent with the previous survey, prior expert recommendations, and multiple clinical practice guidelines, as well as evidence that LAIs reduce relapse risk^26^. The strong endorsement of patient requests is also consistent with the international emphasis on SDM^27^.

LAI initiation at first-episode schizophrenia was only second-line, despite advocacy for early LAI use and meta-analytic evidence suggesting a mortality benefit^28^. This indicates that Japanese specialists remain more conservative toward early LAI use than current international evidence suggests.

When selecting among LAIs (Q12), tolerability and efficacy were both the highest-priority factors, consistent with prior expert consensus and clinical practice guidelines^26,29^. FGA versus SGA was also an important consideration; given meta-analytic evidence of lower adverse-effect risk with SGAs^30^, this again suggests a tolerability-first approach among Japanese specialists. The high ranking of current positive symptom severity likely reflects the symptom-based selection pattern seen in Q1–Q7, as well as differences in efficacy and adverse-effect profiles among individual agents.

Overall, LAI prescribing by Japanese specialists broadly aligns with existing evidence, prior expert consensus, and clinical practice guidelines. However, specialists remained conservative toward early LAI use in first-episode schizophrenia.

In the agitation and medication refusal scenarios (Q13–Q15), OLA and RIS were first-line in both agitation scenarios, whereas FGAs and BZDs were generally lower-ranked. For persistent inpatient medication refusal (Q15), the BLO transdermal patch was the only first-line option.

The Q13 results closely resembled those for excitement and aggression (Q4), with OLA and RIS ranked highest and FGAs and BZDs ranked low. Because evidence on specific antipsychotics for concomitant use during agitation remains limited^31,32^, specialists may have selected OLA and RIS by analogy with the Q4 monotherapy recommendations.

For transient agitation (Q14), the results were broadly consistent with emergency psychiatry guidelines and expert consensus, which favor antipsychotics over BZD monotherapy and recommend non-invasive approaches^33,34^. OLA and RIS likely ranked highly because they combine antipsychotic and sedative effects with better tolerability than FGAs and are available in multiple formulations. The absence in Japan of agents commonly used in Western countries, such as intramuscular lorazepam, may also have influenced these results.

Although LAIs are generally recommended for persistent medication refusal (Q15) in clinical practice^27^, evidence for addressing acute and absolute refusal remains limited. Medication refusal is influenced by poor insight and concerns about adverse effects. Although LAIs and ECT may offer efficacy advantages, their invasive nature may worsen refusal behavior^35^. In contrast, the BLO transdermal patch provides D2 receptor blockade with minimal invasiveness and can be immediately removed if adverse effects occur^36^. This balance of efficacy, safety, and noninvasiveness likely accounts for its high endorsement.

In the treatment-resistant schizophrenia scenarios (Q16–Q19), CLO was the treatment of choice for TRS (Q16), with concomitant ECT as first-line. No first-line consensus was reached on the number of antipsychotics to try before CLO initiation in first-episode TRS (Q17–Q18), regardless of illness severity. All options were second-line. For CRS (Q19), concomitant ECT, waiting 8 or more weeks for response, and increasing the CLO dose to 200 mg or more were first-line.

CLO as the treatment of choice in Q16 is consistent with clinical practice guidelines recommending it after failure of at least two antipsychotics^37^. Concomitant ECT was the next most endorsed option and was rated first-line only as combination therapy, not monotherapy. This is also consistent with guidelines recommending ECT for specific TRS situations and with meta-analytic evidence supporting antipsychotic–ECT combination therapy^38^.

The absence of first-line consensus in Q17 and Q18 likely reflects barriers to CLO access in Japan, despite evidence that earlier CLO introduction may improve treatment response^39^. These barriers include restrictions on prescribing facilities, a requirement for inpatient initiation, written informed consent, psychiatric history review, registration in the Clozaril Patient Monitoring Service (CPMS), and regular hematological monitoring^40,41^. Together, they have limited CLO use in Japan^42^ and may have reduced specialists’ opportunities to gain experience with this drug. Although CLO access is improving^40,41,43^, these changes may take time to become widely established in practice.

For CRS (Q19), international consensus and meta-analysis recommend maintaining CLO plasma levels at 350 ng/mL or higher before adding ECT or other augmentation strategies^44,45^. The first-line ranking of concomitant ECT and waiting 8 or more weeks is therefore consistent with this evidence. However, increasing the CLO dose to 200 mg or more was first-line, whereas plasma level adjustment was only second-line despite meta-analytic support^46,47^. This may reflect the recent introduction of reimbursement for CLO plasma level monitoring in Japan in 2022. Monitoring infrastructure may still be insufficiently established, leading specialists to rely more on dose than on plasma concentration.

In both dose reduction (Q20) and simplification to antipsychotic monotherapy (Q21), adverse effects were the highest-priority factor. Other first-line considerations broadly reflected current clinical stability, relapse-prevention awareness, and patient-level risk assessment. A high number of past episodes was classified as no consensus in both scenarios.

Factors for dose reduction (Q20) were largely consistent with the previous survey, suggesting no major change in overall decision-making criteria. Two notable shifts were observed: the number of previous episodes was downgraded from first-line to no consensus, and past treatment response to second-line. These changes suggest greater emphasis on current clinical status than on past psychiatric history^48^.

The newly added simplification-to-monotherapy scenario (Q21) included all first-line factors from Q20 together with several additional ones. This is consistent with prior evidence that discontinuation of concomitant antipsychotics is more demanding than dose reduction alone^49^. The findings suggest that specialists consider a broader set of conditions necessary for successful monotherapy, including shared decision-making, adherence support, and objective clinical indicators.

This study has several limitations. First, expert consensus provides a lower level of evidence than RCTs and meta-analyses. Second, some scenarios may not have included enough background information for fully informed treatment decisions. These recommendations should therefore be applied with careful consideration of individual patient heterogeneity. Third, generalizability is limited because all participants were Japanese neuropsychopharmacology specialists, and some recommendations may reflect Japan-specific prescribing conditions and drug availability. Stringent regulations governing CLO use in Japan likely influenced the CLO-related recommendations^40,41^. In addition, some survey options, such as perospirone and BLO, are not widely available outside Asia. Fourth, explicit labeling of off-label treatments may have introduced response bias toward lower ratings for those items. Fifth, although the response rate was 44.0%, the institutional distribution of respondents was broadly comparable to that of all invited specialists (e.g., university hospitals: 37.0% vs. 36.6%; psychiatric hospitals: 30.5% vs. 33.1%), suggesting that non-response bias related to institutional affiliation was limited. However, potential differences in clinical experience or prescribing preferences between respondents and non-respondents cannot be ruled out. Finally, the classification of the 9-point scale into three categories and the criteria used to define treatment-line categories were, to some extent, arbitrary, as is common in expert consensus methodology. Future studies employing Delphi methods with iterative feedback may help address this limitation.

Japanese specialists appeared to select pharmacological treatment for schizophrenia by weighing individual clinical characteristics and drug profiles. SGAs, particularly ARI and BRE, were strongly endorsed across multiple clinical situations, reflecting a prescribing approach that placed substantial weight on tolerability and overall efficacy–safety balance. Although these recommendations require further support from future clinical trials, this 2025 consensus provides updated practical guidance for treatment selection and SDM in situations not adequately addressed by existing evidence.

## Supplementary information


Supplementary material


## Data Availability

The datasets used and/or analyzed during the current study are available from the corresponding author on reasonable request.
