# Supplementary material for: Pharmacological treatment of schizophrenia: Japanese Expert Consensus 2025
Source: Schizophrenia (Heidelb). 2026 Jun 2;12(1):70. doi: 10.1038/s41537-026-00770-x (PMC13530244; doi:10.1038/s41537-026-00770-x)
Supplement: Supplementary file 1 [file 41537_2026_770_MOESM1_ESM.docx]

**Supplementary Table 1.** Questionnaire items and response scale

Instructions to Respondents:

In this questionnaire, we would like to inquire about the treatment of patients who have already been diagnosed with schizophrenia. Please rate the level of agreement for each option.

1 2 3 4 5 6 7 8 9

←Disagree Agree→

**Supplementary Figure 1.**

Q1. Which drug would you select when positive symptoms predominate?

Please rate your level of agreement with each drug. If you do not use any of the drugs listed below, please mark 1 for all options. If you use any of them, assign a score of 9 to at least one option.

Abbreviations: CI, confidence interval; SD, standard deviation

**Supplementary Figure 2.**

Q2. Which drug would you select when negative symptoms predominate?

Please rate your level of agreement with each drug. If you do not use any of the following drugs, mark 1 for all options. If you use any of them, assign a score of 9 to at least one option.

Abbreviations: CI, confidence interval; SD, standard deviation

**Supplementary Figure 3.**

Q3. Which drug would you select when depression and anxiety predominate?

Please rate your level of agreement with each drug. If you do not use any of the following drugs, mark 1 for all options. If you use any of them, assign a score of 9 to at least one option.

Abbreviations: CI, confidence interval; SD, standard deviation

**Supplementary Figure 4.**

Q4. Which drug would you select when excitement and aggression predominate?

Please rate your level of agreement with each drug. If you do not use any of the following drugs, mark 1 for all options. If you use any of them, assign a score of 9 to at least one option.

Abbreviations: CI, confidence interval; SD, standard deviation

**Supplementary Figure 5.**

Q5. Which drug would you select when disorganized thinking predominates?

Please rate your level of agreement with each drug. If you do not use any of the following drugs, mark 1 for all options. If you use any of them, assign a score of 9 to at least one option.

Abbreviations: CI, confidence interval; SD, standard deviation

**Supplementary Figure 6.**

Q6. Which drug would you select when cognitive impairment predominates?

Please rate your level of agreement with each drug. If you do not use any of the following drugs, mark 1 for all options. If you use any of them, assign a score of 9 to at least one option.

Abbreviations: CI, confidence interval; SD, standard deviation

**Supplementary Figure 7.**

Q7. Which drug would you select to promote social integration in a patient without noticeable symptoms?

Please rate your level of agreement with each drug. If you do not use any of the following drugs, mark 1 for all options. If you use any of them, assign a score of 9 to at least one option.

Abbreviations: CI, confidence interval; SD, standard deviation

**Supplementary Figure 8.**

Q8. Which drug would you select if a patient is vulnerable to extrapyramidal side effects?

Please rate your level of agreement with each drug. If you do not use any of the following drugs, mark 1 for all options. If you use any of them, assign a score of 9 to at least one option.

Abbreviations: CI, confidence interval; SD, standard deviation

**Supplementary Figure 9.**

Q9. Which drug would you select for a patient with diabetes mellitus?

Please rate your level of agreement with each drug. If you do not use any of the following drugs, mark 1 for all options. If you use any of them, assign a score of 9 to at least one option.

Abbreviations: CI, confidence interval; SD, standard deviation

**Supplementary Figure 10.**

Q10. Which treatment would you select when tardive dyskinesia occurs during antipsychotic treatment?

Please rate your level of agreement with each drug. If you do not use any of the following drugs, mark 1 for all options. If you use any of them, assign a score of 9 to at least one option.

Abbreviations: CI, confidence interval; ECT, electroconvulsive therapy; SD, standard deviation

**Supplementary Figure 11.**

Q11. When would you consider introducing a long-acting injectable antipsychotic?

Please rate your level of agreement with each drug. If you do not use any of the following drugs, mark 1 for all options. If you use any of them, assign a score of 9 to at least one option.

Abbreviations: CI, confidence interval; SD, standard deviation

**Supplementary Figure 12.**

Q12. Which factors would you consider when selecting among long-acting injectable antipsychotics?

Please rate your level of agreement with each drug. If you do not use any of the following drugs, mark 1 for all options. If you use any of them, assign a score of 9 to at least one option.

Abbreviations: CI, confidence interval; SD, standard deviation; FGA, first-generation antipsychotic; LAI, long-acting injectable antipsychotics; SGA, second-generation antipsychotic.

**Supplementary Figure 13.**

Q13. If agitation persists despite antipsychotic monotherapy during acute treatment, which concomitant treatment would you use?

Please rate your level of agreement with each drug. If you do not use any of the following drugs, mark 1 for all options. If you use any of them, assign a score of 9 to at least one option.

Abbreviations: CI, confidence interval; SD, standard deviation

**Supplementary Figure 14.**

Q14. If excitement or agitation temporarily intensifies during inpatient care, which drug would you use for pro re nata administration?

Please rate your level of agreement with each drug. If you do not use any of the following drugs, mark 1 for all options. If you use any of them, assign a score of 9 to at least one option.

*Intravenous injection includes slow intravenous injection and intravenous drip infusion.

*Orally disintegrating tablets are included under standard oral tablets.

Abbreviations: CI, confidence interval; SD, standard deviation

**Supplementary Figure 15.**

Q15. Which treatment would you select for an inpatient with active psychiatric symptoms, poor insight, and persistently refuses medication?

Please rate your level of agreement with each drug. If you do not use any of the following drugs, mark 1 for all options. If you use any of them, assign a score of 9 to at least one option.

*LAI use assumes that oral tolerability has been confirmed previously.

Abbreviations: CI, confidence interval; LAI, long-acting injectable; SD, standard deviation

**Supplementary Figure 16.**

Q16. Which treatment would you select for treatment-resistant schizophrenia?

Please rate your level of agreement with each drug. If you do not use any of the following drugs, mark 1 for all options. If you use any of them, assign a score of 9 to at least one option.

Abbreviations: CI, confidence interval; ECT, electroconvulsive therapy; SD, standard deviation

**Supplementary Figure 17.**

Q17. For first-episode treatment-resistant schizophrenia requiring physical restraint, after how many antipsychotics adequately tried at sufficient doses and for sufficient durations would you consider initiating clozapine?

Please rate your level of agreement with each option. If you do not select any of the options listed below, please mark 1 for all options. If you select any of them, assign a score of 9 to at least one option.

Abbreviations: CI, confidence interval; SD, standard deviation

**Supplementary Figure 18.**

Q18. For the acute phase of first-episode treatment-resistant schizophrenia manageable in an outpatient setting, after how many antipsychotics adequately tried at sufficient doses and for sufficient durations would you consider initiating clozapine?

Please rate your level of agreement with each option. If you do not use any of the following options listed below, mark 1 for all options. If you use any of them, assign a score of 9 to at least one option.

Abbreviations: CI, confidence interval; SD, standard deviation

**Supplementary Figure 19.**

Q19. Which treatment would you select for treatment-resistant schizophrenia that does not respond adequately to clozapine?

Please rate your level of agreement with each treatment. If you do not use any of the options below, mark 1 for all options. If you use any of them, assign a score of 9 to at least one option.

Abbreviations: CLO, clozapine; CI, confidence interval; ECT, electroconvulsive therapy; SD, standard deviation

**Supplementary Figure 20.**

Q20. When pursuing antipsychotic dose reduction in schizophrenia, which of the following factors would you consider?

Please rate your level of agreement with each factor. If you do not consider any of the factors listed below, please mark 1 for all options. If you consider any of them, assign a score of 9 to at least one option.

*The minimum effective dose refers to the lowest dose that produces a clinically effective therapeutic response.

Abbreviations: CI, confidence interval; LAI, long-acting injectable antipsychotic; SD, standard deviation

**Supplementary Figure 21.**

Q21. When pursuing simplification to antipsychotic monotherapy in patients with schizophrenia receiving polypharmacy, which of the following factors would you consider when planning discontinuation of the concomitant antipsychotic?

Please rate your level of agreement with each factor. If you do not use any of the drugs listed below, mark 1 for all options. If you use any of them, assign a score of 9 to at least one option.

Abbreviations: CI, confidence interval; LAI, long-acting injectable antipsychotic; SD, standard deviation
